# Do non-citizens migrate for welfare benefits? Evidence from the Affordable Care Act Medicaid expansion

**DOI:** 10.3389/fpubh.2022.955257

**Published:** 2022-09-30

**Authors:** Hao Guo, Miaomiao Zou

**Affiliations:** ^1^Li Anmin Institute of Economic Research, Liaoning University, Shenyang, China; ^2^School of Economics, Nanjing Audit University, Nanjing, China

**Keywords:** interstate migration, welfare-induce migration, affordable care act, Medicaid expansion, non-citizen immigrants, *H53*, *I13*, *R23*

## Abstract

We explore if low-educated noncitizens, who have a considerably high uninsured rate, internally migrate to states with more generous public insurance benefits. We utilize the state-level variation in accessing Medicaid benefits and employ a difference-in-differences methodology that compares in-migration and out-migration rates of non-citizens in states that adopted Medicaid expansion, both before and after the policy implementation, to the outcomes of non-citizens in states that did not adopt the expansion. We find that interstate in-migration (out-migration) rates of Medicaid expansion states did not increase (decrease) relative to that of non-expansion states after the expansion.

## Introduction

Non-citizen immigrants represent a particularly interesting group for understanding welfare-induced migration both because they have low rate of insurance coverage to begin with and because they constitute a sizeable share of the United States population. In 2019, there were 21.3 million non-citizens in the United States, accounting for approximately 7% of the total population. However, 25% of them were uninsured compared to <9% of citizens (1). Due to limited access to both public and private coverage, the uninsured rate is even higher among low-income non-citizen immigrants. Statistics indicate that more than three quarters of the uninsured non-citizens are low-income [below 200% of the Federal Poverty Level (FPL)], and most of them work in jobs that are less likely to provide health insurance. Will low-income non-citizen immigrants, who are highly uninsured and have a low probability of affording their own coverage, internally migrate out of states with more-stringent rules and into states with more-lenient rules in pursuit of public coverage?

**Figure 1:**
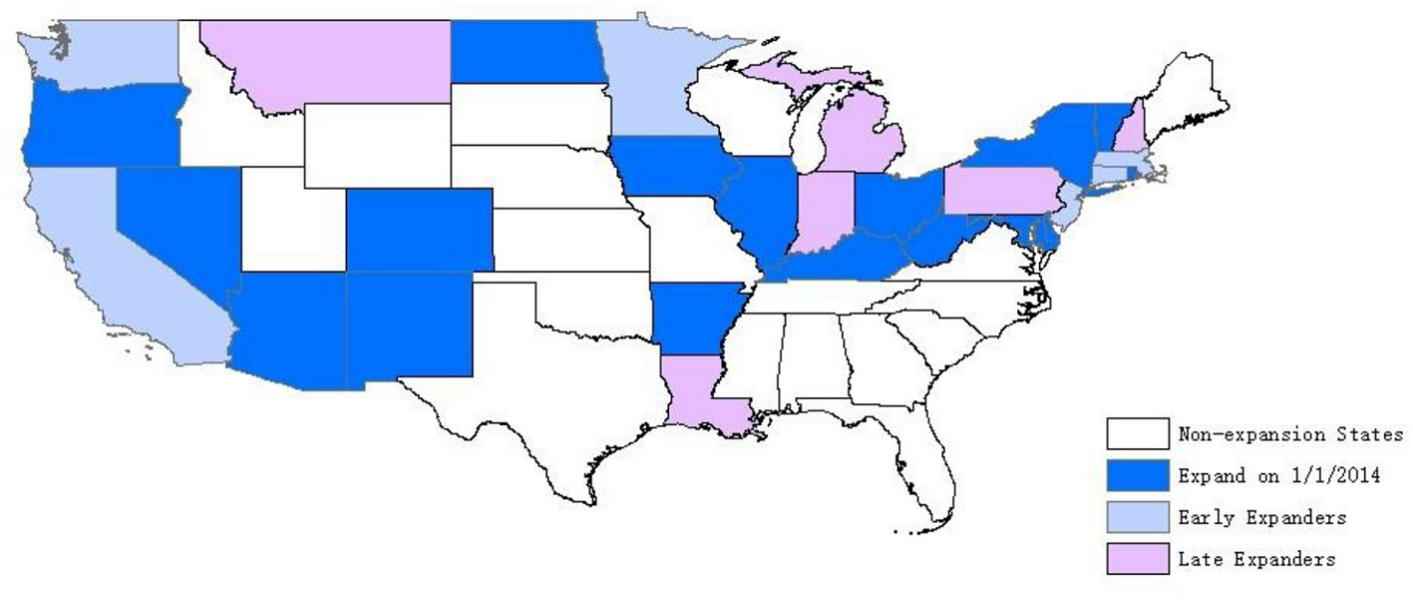
Status of Medicaid expansion decisions, as of 2018. **Source**: The Henry (13).

Medicaid expansion under the Affordable Care Act (ACA) provides us with an opportunity to improve our understanding on this question. The expansion was originally formulated to extend insurance coverage to non-elderly adults with family incomes up to 138% of the FPL at a national level ($30,305 for a family of three in 2021)^1^, but was effectively made a state option by a 2012 Supreme Court ruling. By January 2014, when the expansion was set to begin nationwide, 24 states and the District of Columbia decided to expand their Medicaid program in line with the ACA, whereas the remaining states did not. Later, two additional states implemented during 2014 (MI and NH), three expanded during 2015 (PA, IN and AK), two more expanded in 2016 (LA and MT), as shown in Figure 1^2^. Given that states are not permitted to condition Medicaid eligibility on length of residence, one implication is the possibility that less well-off residents migrate to states that adopted the expansion. One more possibility is that states with expanded Medicaid coverage may result in higher residential satisfaction which would inhibit out-migration.

One strand of the welfare-induced migration literature, largely focused on *international migration*, emphasizes that immigrants make their location decisions at least partly on the basis of the generosity of welfare programs (2). Another strand of the literature explores *intranational migration* within the United States due to variations in public benefits across states. Most of the studies in *intranational migration* focus on the general low-income population (3–6) and conclude with modest welfare migration effects. There is, however, only scant evidence of welfare-induced interstate migration on non-citizen immigrants.

Yasenov et al. (7) is one of the few studies that focus on immigrants. Welfare reform in 1996 barred immigrants who are lawful permanent residents with <5 years of residency in the United States from accessing Medicaid benefits (5-year ban). Later, several states extended coverage to lawfully residing children and pregnant women without a 5-year waiting period. They take advantage of this state level inequalities in eligibility and explore whether these two specific immigrant groups who are excluded from the 5-year ban in some states move across state borders to access public coverage. They find no evidence that the introduction of Medicaid benefits in a specific state was associated with increases in migration of immigrants from other states among the targeted two groups of immigrants.

Non-citizen immigrants' utilization of welfare benefits have been a contentious policy issue for decades. Some believe that “high rates of immigration are straining the health care system to the breaking point” (8). While others claim that immigrants use a disproportionately small share of the nation's health care costs (9, 10) and are paying more than what they are receiving (11, 12). Studying welfare-induced migration is a way of measuring immigrants' response to public benefits. In this paper, we investigate how state variation in accessing Medicaid coverage affected access to insurance coverage and interstate migration of the low-educated non-citizen immigrants. Our general empirical strategy is a difference-in-differences (DD) approach that compares insurance coverage and interstate migration flows of low-educated non-citizen immigrants in states that did and did not expand Medicaid before and after adoption of the policy. To the best of our knowledge, this is the first paper designed to measure the overall immigrant interstate migration response to Medicaid expansion.

The empirical estimates suggest that Medicaid expansion was associated with an increase in insurance rate among low-educated non-citizens by 4.1 percentage points and an increase in Medicaid coverage by about 8.2 percentage points. However, our findings in migration flows indicate that interstate in-migration (out-migration) rate of Medicaid expansion states did not increase (decrease) relative to that of non-expansion states after the expansion, which suggest that the variation in accessing Medicaid coverage did not result in a meaningful effect on interstate movements among the studied sample.

Our study contributes to the literature on welfare-induced migration in several ways. First, we rely on the most recent expansion of Medicaid coverage and focus on the low-educated non-citizen immigrants. Second, we examine the migration responses of non-citizens to and from states with expanded Medicaid eligibility by explicitly exploring both in-migration and out-migration flows. Third, we use several years of data after 2014 to uncover any longer-run effects that may not be visible immediately after the expansion.

## Methods

### Data source and classification of states

We obtain data for the period 2010–2017 from the American Community Survey (ACS) to explore the impact of Medicaid expansion on insurance coverage and interstate migration flows among non-citizen immigrants. The ACS surveys a cross-sectional 1% sample of U.S. households every year. It's large sample size (approximately 3 million observations in all) allows us to focus on a subset of the general population (i.e., non-citizen immigrants) without losing estimation precision. Besides, participation of this survey is mandatory which reduces concerns about sample selection issue^3^. In the ACS, we observe whether a respondent is currently residing in a different state than 1 year prior to interview, as well as the exact state of residence in those two periods^4^.

### Sample selection and classification of states

There are concerns associated with selecting the analysis sample using income since income is potentially affected by the policy and migration^5^. Instead, education is exogenous to the expansion and the common practice is to use low-educated group as a proxy for low-income group in examining effects of means-tested welfare programs. Accordingly, we restrict our main sample to non-citizen immigrants with less than high school education. Using education level to incorporate eligibility reduces the endogeneity-of-sampling issue, however, it may create attenuation bias. For comparison, we present results for low-income sample in a robustness analysis. To capture a broad range of low-educated non-citizens whose interstate migration behavior might plausibly have been affected by the expansion, our primary sample was further restricted to those at the ages of 18–64^6^. To coincide with the 5-year waiting period that qualified non-citizens need to observe before they become eligible for Medicaid coverage (5-year ban), we restrict the sample to non-citizens with at least 5 years of residency in the United States^7^. With these exclusions, the baseline sample includes 305,386 non-citizens^8^.

To classify states into those experienced changes in Medicaid coverage and those not, we rely on Kaiser Family Foundation's (KFF) annual 50 states survey of eligibility rules. One complication with classifying which states into those experienced a change in Medicaid policy (“treated”) and those not (“control”) is that the ACA allows states flexibility to expand Medicaid coverage before 2014, and several states did so to varying degrees. Since the passage of the ACA in 2010, five states (CA, CT, MN, NJ, and WA) and District of Columbia (DC)^9^ have enacted Medicaid expansion that include some or all of the low-income adults who will become eligible for Medicaid, starting in 2014, under the ACA (18, 19). In addition, MA experienced significant policy reforms prior to 2010. Individuals in these states may or may not experience a policy change after 2014 depend on the degree of prior expansion. In the main analysis sample, we consider states that expanded Medicaid coverage by 2014 as the treatment group^10^. The other issue is that there is no deadline for states to decide whether or not to adopt the expansion. As we discussed, most of states expanded on the first day of 2014, but a handful expanded in later years. In the main analysis sample, we exclude states that expanded after the first day of 2014 (MI, NH, PA, IN, AK, LA, and MT). In other words, we only consider states that did not expand Medicaid coverage during the study period as the control group.

Unweighted summary statistics^11^ for the studied sample are calculated and stratified by state Medicaid expansion status (see Table 1). In the primary sample, 64% of the observations are in expansion states, while 36% are in non-expansion states. In terms of demographic differences, characteristics are fairly well-matched, except that non-citizens in non-expansion states tend to have a higher rate of poverty. In non-expansion states, 51% of the low-educated non-citizens has income at or below 138% of the FPL, while it is 47% in Medicaid expansion states. In terms of health insurance coverage, expansion states have relatively higher rate of coverage, mostly coming from Medicaid coverage, though higher rate of employer-sponsored coverage also contributes to the disparity. In terms of labor market outcomes, however, the two sets of states look fairly similar.

**Table 1:** Descriptive statistics (unweighted).

	**Non-expansion states**		**Expansion states**	
	**Mean**	**SD**	**Mean**	**SD**
**A: Demographic variables**
Age	41.31	11.10	42.54	10.91
Female	0.46	0.50	0.48	0.50
Married	0.64	0.48	0.62	0.49
Num. of own children	1.62	1.50	1.64	1.47
Hispanic	0.91	0.28	0.88	0.33
Family income as of FPL	160.65	117.42	171.73	121.78
% with income ≤ 138% of the FPL	0.51	0.50	0.47	0.50
**B: Health insurance coverage**
Uninsured	0.69	0.46	0.50	0.50
Employer-sponsored	0.19	0.39	0.23	0.42
Privately purchased	0.24	0.42	0.26	0.44
Medicaid	0.08	0.26	0.24	0.42
**C: Labor market**
In labor force	0.69	0.46	0.69	0.46
Unemployed	0.07	0.26	0.10	0.30
Ln (hours)	3.60	0.40	3.58	0.42
Fulltime	0.88	0.32	0.86	0.34
*Obs*.	109,162		196,224	
**D: In-migration rate**
Cross-state migration	0.0123	0.11	0.0068	0.0825
Cross E/NE state migration	0.0065	0.0804	0.0023	0.0477
*Obs*.	109,162		196,224	
**E: Out-migration rate**
Cross-state migration	0.0099	0.0988	0.0082	0.0901
Cross E/NE state migration	0.0041	0.0639	0.0036	0.0600
*Obs*.	108,898		196,488	

**Source:** American Community Survey (2010–2017).

Sample is limited to non-citizens with less than high school education at ages 18–64. Each cell in panels A-C reports the sample mean of the variable indicated, among noncitizens in the baseline sample with current state in non-expansion states (column 1) or in expansion states (column 2). Panels D and E report the average in-migration and out-migration rate for the studied sample. Cross-state migration means moved across a state border line. Cross E/NE state migration means moved across a state border line between an expansion state and a non-expansion state. FPL is federal poverty level.

### Interstate migration: In-migration and out-migration

The expansion of Medicaid coverage could have attracted non-citizens from non-expansion states to move in or persuaded those in expansion states who might have considered leaving to stay put or change destination, after the expansion. Therefore, the most powerful measure to detect interstate migration effects of the Medicaid expansion is to investigate the relative changes in migration rate between expansion and non-expansion states. Accordingly, we define two binary variables (i.e., in-migration and out-migration) to estimate the potential effects.

It is motivated in part by Goodman (20) who explores whether non-expansion-to-expansion migration increased, relative to the increase in expansion-to-non-expansion migration among low-income population. He shows that migration from non-expansion states to expansion states did not increase relative to migration in the reverse direction. One of our outcome variables, out-migration, is similar to his specification, which is defined from the perspective of a respondent's original state of residence (12 months prior to interview). It equals one if a person had moved out of an expansion state and moved into a non-expansion state (out-migration for expansion states) or moved out of a non-expansion state and moved into an expansion state (out-migration for non-expansion states) in the last 12 months. However, this specification only explores one aspect of the potential migration effect of Medicaid expansion.

Migration is not only affected by factors that push individuals to migrate out (perspective from state-of-origin), but also by factors that pull individuals to move in (perspective from state-of-destination). Based on this logic, when benefits are more favorable in some states, such conditions are expected to create an incentive for potential beneficiaries to migrate in. Earlier studies also show the concern of that characteristics in destination regions may correlate with both welfare policy and immigration pattern (21, 22). Accordingly, we define another outcome variable, in-migration, as from the perspective of state-of-destination (current state of residency). And we assume that a state's in-migration rate is affected by current state's economic conditions. Following Kaushal (23), we introduce state-of-destination fixed effect to control for unobserved time-invariant destination characteristics. If the expansion of Medicaid coverage changes interstate migration rate, then we should expect an increase in in-migration rate and a decrease in out-migration rate. We will discuss further in *Statistical Analysis*.

Swartz and Sommers (5) use “cross-state in-migration and out-migration” to examine the potential interstate migration effect of public insurance expansions in AZ, ME, MA, and NY. They define “cross-state in-migration” as whether a person had moved into an expansion or a non-expansion state from another state in the previous years and define “cross-state out-migration” as whether a person who had been living in a non-expansion state or expansion state had moved to another state in the previous year. However, “cross-state migration” includes not only migrations from expansion states to non-expansion states or from non-expansion states to expansion states but also migrations between expansion states and between non-expansion states. Immigration flows either between expansion states or between non-expansion states should not be counted as welfare-induced migration.

We present average migration rate for both definitions of “in-migration” and “out-migration” in panels D and E of Table 1 for comparison. The first row of panels D and E shows the mean of cross-state in-migration and out-migration rate. The second row shows the mean of our measurement of in-migration and out-migration rate (i.e., moved from an expansion state to a non-expansion state or in a reverse direction). On average, in our sample period, cross-state in-migration rate averaged 0.88 percent each year (1.23 percent in non-expansion states and 0.68 percent in expansion states), and out-migration averaged 0.88 percent^12^ (0.99 percent in non-expansion states and 0.82 percent in expansion states). Only 30–50 percent of the non-citizens who migrated across states, or 0.38 percent of the sample, said that they had changed their state of residency from an expansion state to a non-expansion state or in a reverse direction.

**Figure 2:**
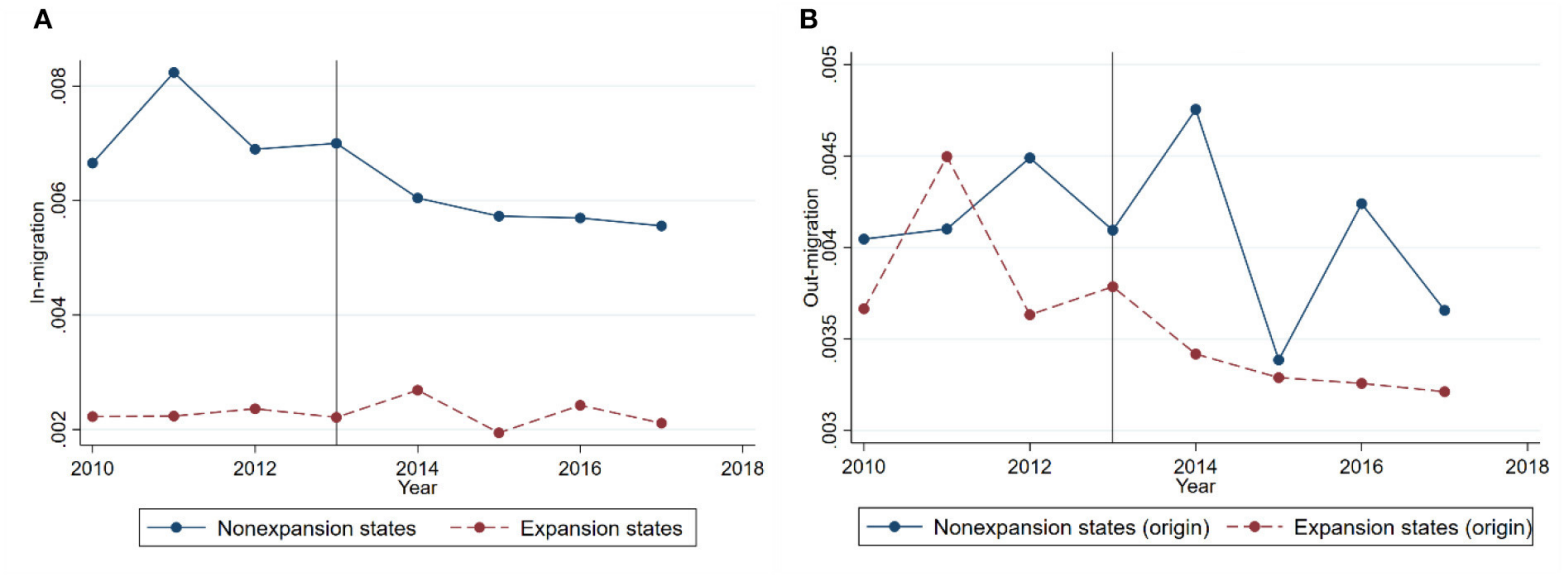
**(A)** In-migration. **(B)** Out-migration. Yearly average in-migration and out-migration rates for the main analysis sample. **Sources**: 2010 to 2017 American Community Survey. Annual means of in-migration and out-migration rates for expansion and non-expansion states are plotted in the figure. The sample consists of less than high school educated non-citizens at ages 18–64 with more than 5 years of residency in the United States.

To understand broad patterns of migration flows between expansion and non-expansion states in the analysis sample, we plot annual means of in-migration and out-migration rates for Medicaid expansion and non-expansion states in Figure 2. The left figure shows that in-migration rate of non-expansion states decreases relative to that of expansion states after the expansion. The right figure shows that the average percent of non-citizens moving out of expansion states decreases relative to the average percent of non-citizens moving out of non-expansion states, after the expansion. However, the difference in changes is relatively small. Notably, both in-migration and out-migration rates are generally higher in non-expansion states than in expansion states, both before and after the expansion.

### Statistical analysis

#### Health insurance coverage

The effect of Medicaid expansion in reducing un-insurance rate among the general low-educated population has been well documented in the health economics literature [(24, 25) to name a few]. However, the effect on low-educated non-citizens remain underexplored. Therefore, we first investigate if the expansion of Medicaid coverage associates with changes in insurance coverage of low-educated non-citizens before we formally explore the migration effects.

The ACS asked whether an individual had health insurance coverage as well as the type of coverage at the time of interview. Based on the survey questions, we defined four insurance coverage indicator variables. One was a dummy variable and denotes lack of any type of coverage (i.e., uninsured). The other three were indictors for a specific type of coverage, including privately purchased^13^, employer-sponsored and Medicaid. We employ a DD research design and compare changes in health insurance coverage of low-educated noncitizens in states that did and did not expand Medicaid coverage before and after adoption of the policy. The DD approach could cancel out the effect of common factors that affect both the treatment and control groups and isolate the effect of Medicaid expansion. We obtain the DD estimates of the ACA Medicaid expansion by running a regression of the following form on our outcome variables^14^:


(1) $$\begin{array}{l} Y_{ist}=\beta_{0}+\beta_{1}Expan_{is}\times Post_{t}+X\gamma +\eta_{s}+\theta_{t}+\varepsilon_{it} \end{array}$$

Equation (1) indicates that the health insurance coverage, for example, Medicaid, of noncitizen “*i*” in state “*s*” and year “*t*” depends on the interaction of an indicator of whether a noncitizen “*i*'*s*” current state of residency is in the treatment group with a time dummy that equals one after year 2014 (*Expan*_*is*_×*Post*_*t*_), and demographic characteristics and state-level variables (*X*). Demographic characteristics include age, age squared, gender and marital status. State-level variables include unemployment rate [taken from the (26)] and the annual average number of weeks of unemployment insurance (UI) benefits available [adopted from Farber and Valletta (27) and Valletta (28)^15^]. A full set of state-of-destination and year dummies, η_*s*_ and θ_*t*_, are also included in the equation to account for unobserved state-level heterogeneity and common time trends in the outcomes across states. Standard errors are clustered at state-of-destination level to adjust for within-state correlation over time.

#### In-migration and out-migration


(2) $$\begin{array}{l} In\text{-}migration_{ist}\;=\;\beta_{0}+\beta_{1}Expan_{is}\times Post_{t}+X\gamma +\eta_{s}+\theta_{t}\\ \;+\varepsilon_{it} \end{array}$$

the dependent variable, In-migration_*ist*_, equals 1 for a noncitizen *i* who have moved from a non-expansion state to an expansion state or in a reverse direction, in the 12 months prior to interview in year *t*, otherwise equals to zero. *Expan*_*is*_ × *Post*_*t*_ is the interaction of an indicator of whether a respondent's state-of-destination (current state) is an expansion state with a time dummy that equals one after year 2014. X includes the same set of demographic characteristics and state-level variables as in equation (1). Standard errors are clustered at the state-of-destination level. Therefore, coefficient β_1_ captures the relative change (expected to be weakly positive) between inflows in these two directions after 2014 relative to that difference in earlier years.


(3) $$\begin{array}{lll} Out\text{-}migration_{ist}\;=\;\beta_{0}+\beta_{1}Expan_{origin_{{}_{is}}}\times Post_{t}+X_{origin}\gamma & & \\ \;+\eta_{origin_{s}}+\theta_{t}+\varepsilon_{it} & & \end{array}$$

*Expan*_*origin*_ is a dummy variable that equals one if a respondent's state-of-origin is an expansion state. *X*_*origin*_ includes the same set of controls as in equation (1) but refers to last year. _η_*origin*_*s*_ is a set of state-of-origin dummies. Standard errors are clustered at the state-of-origin level. Therefore, coefficient β_1_ captures the relative difference (expected to be weakly negative) of out-migration rates between expansion and non-expansion states after 2014 relative to that difference in earlier years.

## Results

### Health insurance coverage

We begin the discussion of results with the effect of Medicaid expansion on insurance coverage, which is classified into Medicaid, private insurance, employer sponsored insurance and uninsured. Regression estimates in Table 2 indicate that the expansion of Medicaid coverage was associated with 4.1 percentage points (from a base of 57.59 percent) decrease in un-insurance rate, 2.31 percentage points (from a base of 21.71 percent) decrease in employer-sponsored coverage, and 3.81 percentage points (from a base of 23.84 percent) decrease in private coverage for low-educated non-citizens in expansion states relative to their counterparts in non-expansion states. The changes were driven entirely by an increase in Medicaid coverage (8.18 percentage points). All estimates are statistically significant. The decrease in private insurance and increase in Medicaid coverage suggest some amount of crowd-out of private for public insurance. In short, our findings in health insurance coverage are in line with published evidence, which found robust evidence that the expansion of Medicaid coverage is associated with significant increase in insurance rate.

**Table 2:** Changes in health insurance coverage.

	**Medicaid**	**Private purchase**	**Employer-sponsored**	**Uninsured**
Expand × post 2014	0.0818***	−0.0381***	−0.0231***	−0.0410**
	(0.0094)	(0.0108)	(0.0060)	(0.0154)
Mean of dep. var. in expansion states before 2014	[0.1867]	[0.2384]	[0.2171]	[0.5759]
State fixed effect and year fixed effect	Yes	Yes	Yes	Yes
*Obs*.	305,386	305,386	305,386	305,386

**Source**: American Community Survey (2010–2017).

Estimates report coefficients on interaction term between an indicator for whether the state is an expansion state and an indicator for whether the year is after 2014. Sample used in this analysis is limited to non-citizen immigrants between ages 18 and 64. Regressions are adjusted using indictors for state, year, age, age squared, gender, and marital status. State level variables include the unemployment rate and the annual average number of weeks of unemployment insurance benefits available. Regressions are weighted by the ACS sample weights. All standard errors (parentheses) are clustered on current-state level. Mean of dependent variables in the set of expansion states before 2014 are reported in brackets.

^*^ p < 0.1;

^**^ p < 0.05;

^***^ p < 0.01.

The estimates (see Appendix Table A1) for the low-income sample (at or below 138% of the FPL^16^) are very similar to those for the low-educated sample (Table 2), although slightly larger. For example, the 2014 Medicaid expansion was associated with a 10.07 percentage point increase in Medicaid coverage, a 5.51 percentage point decrease in uninsured, and a 4.46 percentage point decrease in private insurance. All estimates are statistically significant. Overall, the estimates suggest a slightly higher rate of crowd-out of private for public insurance than in the low-educated sample.

### In-migration and out-migration: Baseline results

In this subsection, we address our primary question: whether the expansion of Medicaid coverage associates with changes in interstate migration patterns among the low-educated noncitizen immigrants. To explore this question, we estimate equations (2) and (3) and report the corresponding estimates in Table 3^17^.

**Table 3:** Changes in in-migration and out-migration rates: Main results.

	**Model 1**		**Model 2**		**Model 3**	
	**In-migration**	**Out-migration**	**In-migration**	**Out-migration**	**In-migration**	**Out-migration**
Expand × post 2014	0.0001	0.0005	−0.0004	0.0003	0.0007	−0.0007
	(0.0010)	(0.0008)	(0.0010)	(0.0008)	(0.0024)	(0.0015)
Mean of dep. var. in expansion states before 2014	[0.0023]	[0.0023]	[0.0023]	[0.0023]	[0.0023]	[0.0023]
Demographic controls	Yes	Yes	Yes	Yes	Yes	Yes
State fixed effect and year fixed effect	Yes	Yes	Yes	Yes	Yes	Yes
State-level controls	No	No	Yes	Yes	Yes	Yes
State specific linear trends	No	No	No	No	Yes	Yes
*Obs*.	305,386	305,386	305,386	305,386	305,386	305,386

**Source**: American Community Survey (2010–2017).

Sample used in the analysis is limited to non-citizen immigrants between ages 18 and 64 with education level less than high school. Estimates report coefficients on the interaction term of equations (2) and (3). Regressions are adjusted using indictors for state, year, age, age squared, gender, and marital status. State level variables include the unemployment rate and the annual average number of weeks of unemployment insurance benefits available. Regressions are weighted by the ACS sample weights. All standard errors (parentheses) are clustered on current-state level for in-migration equations and origin-state level for out-migration equations. Mean of dependent variables in expansion states before 2014 are reported in brackets. See Appendix Table A3 for a full set of results.

^*^ p < 0.1;

^**^ p < 0.05;

^***^ p < 0.01.

We first estimate a model that without including state level variables and present the estimates in Model 1. In model 2, we further include the state-level unemployment rate and the annual average number of weeks of unemployment insurance (UI) benefits available. Buchmueller et al. (29) argue that part of the reason that individuals migrate to expansion states is because they can spend more time on unemployment and search for a better job. This raises a concern of that state-level decisions in expanding Medicaid coverage may correlate with state-level economic conditions. The other concern is that Medicaid expansion may correlate with changes in other welfare programs that also affect migration. Most of the states reduced their UI duration back to normal level of 26 weeks when the Emergency Unemployment Compensation program^18^ was terminated at the end of 2013, which corresponds exactly to when Medicaid expansion took effect (on Jan 1, 2014). As discussed by Buchmueller et al. (29), the reduction in UI availability could offset and hence bias the estimated impact of Medicaid expansion. Statistics also show that, on average, expansion states tend to be more generous on UI benefits than non-expansion sates, and a number of non-expansion states reduced their normal UI duration below 26 weeks after 2014. Thus, it is important to control for the generosity of UI benefits. In model 3, we further include state-specific linear trends to control for any difference in the trends of interstate migration between expansion and non-expansion states^19^.

The estimates in model 1 indicate that Medicaid expansion was associated with an increase in in-migration rate of 0.01-percentage-point and an increase in out-migration rate of 0.05-percentage-point, however both estimates are statistically insignificant. Model 2 includes state unemployment rate and UI durations, and the results indicate that Medicaid expansion was not associated with a change either in in-migration or out-migration. Model 3 adds state-specific trends and results show no potential difference in migration trends between expansion and non-expansion states. In brief, the regression estimates suggest no statistically significant changes in in-migration and out-migration flows resulting from Medicaid expansion^20^.

### Parallel trend assumption

This estimation strategy relies on a standard parallel trend assumption, which means in the absence of the Medicaid expansion changes in in-migration and out-migration rates would be the same in the treatment and control groups. To assess the validity of this assumption, we re-estimate the models that produced the main results, but allow the treatment indicator to differ by every year instead of just pre- and post-2014. The parallel trend assumption implies that all pre-2014 interactions between the treatment indicator and the year dummies are zero.

**Figure 3:**
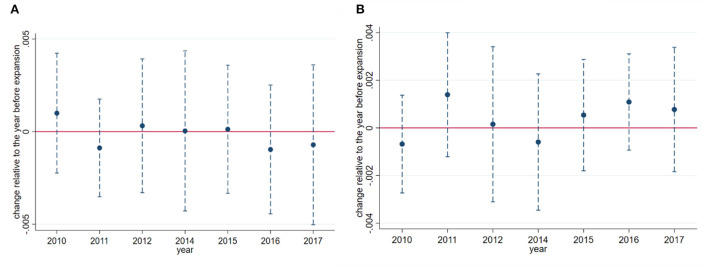
**(A)** In-migration. **(B)** Out-migration. Event study of in-migration and out-migration rates among non-citizens. **Sources**: Authors' difference-in-differences (DD) estimates from the 2010 to 2017 American Community Survey. Coefficients (blue dots) and 95% confidence intervals (blue vertical dashed lines) from DD regressions are plotted in the figure (year 2013 is omitted). The sample consists of less than high school educated non-citizens at ages 18–64 with >5 years of residency in the United States. Regressions are adjusted using indictors for state, year, age, age squared, gender, marital status and two state-level variables. States and year fixed effects are also included. All standard errors (parentheses) are clustered on state. Regressions are weighted by the ACS sample weights.

Figure 3 provides a visual summary of the preexisting trends in in-migration and out-migration rates. It shows that non-expansion-to-expansion migration and expansion-to-non-expansion migration was following a reasonably parallel trend before the expansion. Indeed, none of the coefficients significant at conventional level and they are not joint significantly different from zero in years prior to 2014 (*p*-value of 0.5981 for in-migration and 0.5680 for out-migration). Therefore, the parallel trend assumption holds in our study.

### Border analysis: Short distance move

Several papers claim that individuals are migrating as short a distance as possible to obtain higher welfare benefits (3, 4). The ACS identifies localities known as Public Use Microdata Areas (PUMAs)^21^–approximately 2,300 areas of at least 100,000 people nested entirely within a state. We separate noncitizens within a state into different regions based on their PUMAs and define two sets of variables to examine this possibility. We report the results in Table 4.

**Table 4:** Changes in in-migration and out-migration rates: Short distance move.

	**In-migration**	**Out-migration**
**A: PUMA within a certain distance to state border**		
PUMAs that straddle state border	0.0006	0.0018
	(0.0038)	(0.0027)
*Obs*.	39,505	37,380
< 50 km from state border	0.0005	0.0021
	(0.0020)	(0.0013)
*Obs*.	76,932	69,859
< 100 km from state border	−0.0001	0.0016
	(0.0019)	(0.0012)
*Obs*.	112,521	102,281
**B: PUMA within a certain distance to expansion/non-expansion state border**		
PUMAs that straddle E/NE state border	0.0007	0.0005
	(0.0084)	(0.0044)
*Obs*.	15,342	14,566
< 50 km from E/NE state border	0.0009	−0.0003
	(0.0033)	(0.0019)
*Obs*.	17,546	15,861
< 100 km from E/NE state border	−0.0018	0.0006
	(0.0039)	(0.0023)
*Obs*.	36,203	33,015
< 200 km from E/NE state border	−0.0013	0.0010
	(0.0032)	(0.0019)
*Obs*.	63,375	57,638

**Source:** American Community Survey (2010–2017).

Estimates report coefficients of the interaction term of Equations (2) and (3). Sample used in this analysis is limited to non-citizen immigrants between ages 18 and 64 with education level less than high school. Regressions are adjusted using indictors for state, year, age, age squared, gender, marital status and two state-level variables. Regressions are weighted by the ACS sample weights. All standard errors (parentheses) are clustered on current-state level for in-migration equations and origin-state level for out-migration equations.

^*^ p < 0.1;

^**^ p < 0.05;

^***^ p < 0.01.

First, we define “state border” as a border between two states. In the first row of panel A, we restrict the sample to individuals in PUMAs (Current-PUMA for in-migration and PUMA-origin for out-migration) that straddle a border between two states. However, only 13% of the sample satisfies this restriction. Therefore, in the remaining rows, we restrict the sample somewhat less stringently. In particular, we keep the sample to those in PUMAs with centroids within 50 km and 100 km to a state border, respectively. In brief, estimates in panel A of Table 4 are quite close to zero in both in-migration and out-migration equations in all specifications.

Second, we define “E/NE state border” as a border between an expansion state and a non-expansion state. We restrict the sample to individuals in PUMAs (Current-PUMA for in-migration and PUMA-origin for out-migration) that straddle an E/NE state border. Of the studied sample, 5% of the observations in PUMAs that straddle a border between an expansion and a non-expansion state. We, therefore, test the result by restrict the sample to those reside in PUMAs with centroids within 50 km, 100 km and 200 km to an E/NE state border. Regression estimates in panel B tell a similar story as the estimates in panel A: there is no association between Medicaid expansion and interstate migration^22^.

### Subsample analysis

If Medicaid expansion changes migration behavior, it should be easier to identify such an effect among a group with relatively higher demand for health care or with a relatively higher geographic mobility. We conduct a range of tests to investigate this possibility.

First, prior to 2014, unemployed individuals are roughly three times as likely to be uninsured as employed workers (29). In other words, employed workers have a higher probability of covered through employer sponsored insurance which in turn decreases their demand for other types of insurance coverage. In comparison, unemployed individuals may be particularly reliant on public insurance. Molly et al. (30) show that individuals are more likely to have moved across state if they were unemployed in the previous year. Therefore, we restrict the sample to those with no employment to test if they are more responsive to the expansion of Medicaid^23^.

Second, prior to the ACA, most states had income eligibility for Medicaid that were more stringent for adults without dependent children than for adults with dependent children^24^. Also, it is much easier for individuals to migrate without dependent children than with dependent children. Therefore, we hypothesize that Medicaid expansion have stronger effects on non-citizens without dependent children than on parents. Accordingly, we limit the sample to those without dependent children to investigate this possibility.

Third, compared with established immigrants who have stronger social networks, new immigrants are more flexible to move. To test this possibility, we restrict the sample to those with <10 years of migration.

Forth, single men have the highest geographic mobility rate in our sample (1.6 percent moved across states and 0.6 percent moved across expansion/non-expansion states). Therefore, we restrict the sample to the most mobile group to investigate if they are more responsive to the expansion.

We show the results of subgroup analysis in Table 5. While the signs of the estimates are generally consistent with the welfare magnet theory (more in-migration and less out-migration), none of the estimates are statistically significant. In short, results are consistent with a null effect of Medicaid-induced migration, even among sub-populations with expected higher demand for insurance coverage or with greater graphical mobility.

**Table 5:** Changes in in-migration and out-migration rates: Subsamples with relatively higher demand and/or higher geographic mobility.

	**In-migration**	**Out-migration**
Unemployed	−0.0002	−0.0001
	(0.0022)	(0.0013)
*Obs*.	113,376	93,535
Without dependent children	0.0009	−0.0003
	(0.0014)	(0.0013)
*Obs*.	142,994	142,994
Years of migration (>5 and ≤ 10)	0.0007	−0.0015
	(0.0025)	(0.0026)
*Obs*.	56,773	56,773
Single men	0.0003	0.0005
	(0.0021)	(0.0017)
*Obs*.	61,891	61,891

**Source:** American Community Survey (2010–2017).

Estimates report coefficients of the interaction term of equations (2) and (3). Sample used in this analysis is limited to non-citizen immigrants between ages 18 and 64 with education level less than high school. Regressions are adjusted using indictors for state, year, age, age squared, gender, marital status and two state-level variables. Regressions are weighted by the ACS sample weights. All standard errors (parentheses) are clustered on current-state level for in-migration equations and origin-state level for out-migration equations.

^*^ p < 0.1;

^**^ p < 0.05;

^***^ p < 0.01.

### Robustness checks

Robustness analyses consist of several steps. First, we test the sensitivity of our results to alternative definitions of treatment and control groups and present the estimates in panel A of Table 6. And then, we deal with sample selection issue and show the results in panel B. Finally in panel C, we present several robustness checks experiment with dropping groups of individuals with potentially ambiguous treatment statuses.

**Table 6:** Changes in in-migration and out-migration rates: Alternative ways of handing the sample.

	**In-migration**	**Out-migration**	**In-migration**	**Out-migration**	**In-migration**	**Out-migration**
**A: Alternative identification strategies**						
	Drop early expanders^a^		Include all states^b^		Allow variation in treatment timing^c^	
Expand × post2014	−0.0004	−0.0002	−0.0002	−0.0002	−0.0015	−0.0006
	(0.0011)	(0.0009)	(0.0012)	(0.0009)	(0.0016)	(0.0021)
*Obs*.	179,009	179,009	313,830	313,830	313,830	313,830
**B: Alternative sample selections**						
	Income ≤ 138%FPL		Edu < HS and Income ≤ 138% FPL		Income ≤ 100% FPL	
Expand × post2014	−0.0021	0.0017*	−0.0012	0.0009	−0.0013	0.0006
	(0.0016)	(0.0008)	(0.0016)	(0.0010)	(0.0019)	(0.0011)
*Obs*.	289,658	289,658	147,278	147,278	193,871	193,871
**C: Restricted samples**						
	Exclude ages 18–25		Exclude year 2013		Exclude undocumented immigrants	
Expand × post 2014	−0.0003	0.0001	−0.0005	0.0003	0.0009	−0.0010
	(0.0009)	(0.0008)	(0.0010)	(0.0009)	(0.0011)	(0.0013)
*Obs*.	286,002	286,002	266,353	266,353	116,863	116,863

**Source:** American Community Survey (2010–2017).

Estimates report coefficients of the interaction term of equations (2) and (3) by using model 2. Regressions are adjusted using indictors for state, year, age, age squared, gender, marital status and two state-level variables. Regressions are weighted by the ACS sample weights. All standard errors (parentheses) are clustered on current-state level for in-migration equations and origin-state level for out-migration equations.

^*a*^Early expanders include: CA, CT, MN, NJ, WA, and DC.

^*b*^Late expanders include: MI, NH, PA, IN, AK, LA, and MT.

^*c*^We use cross-state in-migration and out-migration as our outcome variables under this specification. We cannot use cross expansion/non-expansion state migration because the set of expansion states gets larger and the set of non-expansion states gets smaller along with more states counted as expansion states. Mechanically, this decreases in-migration rate in the set of expansion states and increases in-migration rate in the set of non-expansion states.

^*^ p < 0.1;

^**^ p < 0.05;

^***^ p < 0.01.

Panel A shows results of using alternative classifications of treatment and control groups. First, it is reasonable to expect that the effect of the 2014 ACA Medicaid expansion is smaller in states with prior expansions. Therefore, we restrict the treatment group to states without Medicaid expansion prior to January 2014. In this test, we still consider states that did not expand Medicaid coverage during our study period as the control group. Results in column 1 of panel A indicate that our results are not sensitive to the inclusion of “early expanders.” Second, as mentioned, in the main specification, we exclude states that expanded Medicaid after the first day of 2014 (MI, NH, PA, IN, AK, LA, and MT). In robustness, we employ two methods to take consideration of the “late expanders.” The simpler way to address this problem is considering the “late expanders” as “treated” (column 2). Furthermore, to exploit variations across groups of units that received treatment at different times, we allow variations in treatment timing (column 3)^25^. Results in both columns are not significantly different from those of the models that excluded the “late expanders.”

Panel B shows the results of using alternative samples. We present results for non-citizens with incomes up to 138% of the FPL in column 1 of panel B. We also present the results for the sample of non-citizens who qualify for both income restriction (at or below 138% of the FPL) and education restriction (less than high school educated) in column 2 of panel B. Regression estimates are close to zero in both selections, however, estimate in income-based selection is significant at 10 percent level for out-migration and the sign of the estimated coefficient is opposite as what we expected. One problem of using income threshold to select sample is that people may self-select into the treatment group. For example, individuals in expansion states may adjust their income to obtain Medicaid benefits. This would lead to a bias against finding welfare-induced migration and may explain the results obtained using income-based section.

To subsidize health insurance for those too poor to afford it, the ACA also introduces health insurance exchanges for adults with family incomes between 100–400% of the FPL. In non-expansion states, qualifying adults with incomes in the range of 100–138% of the FPL are eligible for federal subsidies in the form of premium tax credits to purchase through health insurance exchanges, which is somewhat less generous^26^ than Medicaid coverage. The ACA was written anticipating that all states would expand Medicaid coverage, so it limits subsidies to individuals with income below the poverty line. As a result, individuals with income below 100% of the FPL fall into what has been dubbed the Medicaid “coverage gap” if they reside in states opting not to expand Medicaid eligibility. In other words, there is a sharp variation across states in benefits for adults with family incomes below the federal poverty level. We, therefore, restrict the sample to non-citizens with incomes below 100% of the FPL to test the potential effects. Results in the last column of panel B indicate that this group of non-citizens are not statistically significant affected by the expansion.

Panel C report results of using several restricted samples. First, the ACA includes a provision requiring insurers to allow children to stay on their parent's health insurance plans until their 26th birthday, beginning in September 2010. Generally, evidence in the literature indicates that this provision led to a significant increase in insurance coverage among the 18 to 25-year-olds (19, 31). To reduce the possibility of confounding from this earlier provision, we re-estimate our sample by excluding individuals at ages 18–25. Second, as argued by Goodman (20), individuals might migrate in anticipation of expansion. Therefore, moves reported in the 2013 survey could also be part of the treatment effect. Following Goodman (20), we drop observations from the 2013 survey to test the robustness of our main results. Results in columns 1 and 2 indicate, neither restriction yields results that are meaningfully different from the baseline specification. Third, undocumented immigrants are prohibited from receiving public benefits and include them in the sample may lead the estimates biased toward zero. However, like other official datasets representing the U.S. population, the ACS does not share information on the legal status of immigrants. To identify the undocumented immigrants, we employ a “residual methodology” following Passel and Cohn (32) and Borjas (33)^27^. Results presented in the last column of panel C indicate an insignificant effect of Medicaid expansion on either in-migration or out-migration of the documented noncitizens^28^.

### Interstate migration for pregnant women and child immigrants

Table 7 provides additional DD estimates testing the robustness of our findings. As discussed above, we restrict the main sample to those with more than 5 years of U.S. residency because quantified immigrants must wait 5 years before they are eligible for Medicaid benefits. However, non-US-born children (age <18 years) and pregnant women are excluded from the 5-year ban in some states. The two groups of immigrants might be incentivized to move because they would qualify for the expanded coverage^29^. In robustness, we use this state-level variation in extending Medicaid benefits to these two specific groups of immigrants to estimate if they are more likely to move to states with expanded coverage. We present the results for non-citizen immigrants with at least one non-US-born dependent children (age <18 years) in the left column of Table 7 and present the estimates for women at reproductive age (18–49 years) in right column of Table 7. Neither group of the immigrants experienced significant movement across states in response to the expanded benefits.

**Table 7:** Changes in in-migration and out-migration rates: Interstate migration for pregnant women and child immigrants.

	**With at least one non-US-born dependent children (age**<**18 years)**		**Women at reproductive age (18**–**49 years)**	
	**In-migration**	**Out-migration**	**In-migration**	**Out-migration**
Expand × post 2014	−0.0222	−0.0111	−0.0037	−0.0359
	(0.0210)	(0.0670)	(0.0058)	(0.0940)
*Obs*.	9,464	9,464	28,599	28,599

**Source:** American Community Survey (2010–2017).

Sample used in this analysis is limited to noncitizen immigrants with education less than high school and US residence less than 5 years. Regressions are adjusted using indictors for state, year, age, age squared, gender, marital status and two state-level variables. Regressions are weighted by the ACS sample weights. All standard errors (parentheses) are clustered at current-state level for in-migration equations and origin-state level for out-migration equations.

^*^ p < 0.1;

^**^ p < 0.05;

^***^ p < 0.01.

## Discussion and limitation

Following the passage of the Affordable Care Act (ACA) and the subsequent 2012 Supreme Court decision, some states elected to offer Medicaid coverage to adults with incomes up to 138% of the FPL while others did not. The expansion of Medicaid coverage has the potential to benefit the low-educated non-citizens by providing new coverage pathways for those who are in need. As policy makers continue to debate access to public benefits for non-citizen immigrants, it is important to generate evidence as to whether the expansion of Medicaid coverage shapes interstate migration flow of the more than 4 million Medicaid-eligible non-citizens residing in the United States. In this paper, we investigate whether post-ACA Medicaid coverage differences play a role in insurance coverage and interstate migration flow of the low-educated non-citizens.

Despite the individual-level analyses based on DD models indicate that the 2014 Medicaid expansion was associated with statistically significant increases in insurance coverage rate among the low-educated non-citizens, there is little evidence supporting an increase in in-migration rate or a decrease in out-migration rate in expansion states relative to that of non-expansion states. In other words, states that consider expanding Medicaid coverage are unlikely to experience large increases in migration from other states. We also find no evidence of Medicaid-induced migration when we narrow our analysis to the border PUMAs of expansion and non-expansion states. To home in on the groups with the greatest incentive to migrate, we focus on recently unemployed individuals, childless adults, those within 5 to 10 years of immigration, and single men. We end up with same conclusion that they are no more likely to migrate to benefits providing states. Overall, our findings are generally consistent with welfare-induced migration literature, which has not found robust evidence for interstate migration by potential beneficiaries to states with more generous social welfare programs.

As of 2022, 12 states have not expanded Medicaid coverage. Despite the large increases observed in coverage rate, it remains unclear whether any of the remaining states will adopt the expansion in the near future. States in considering of expanding Medicaid coverage may take welfare-induced migration into account. With an estimated average benefit of $5,500 for the newly eligible in 2014^30^, the gain to a migrant from a non-expansion state to an expansion state could potentially be quite large. However, our preferred estimates indicate that there was little to no net impact of the Medicaid expansion on either in-migration or out-migration. Our findings suggest it is unlikely that low-educated noncitizens migrate to Medicaid expansion states to obtain public coverages. Therefore, interstate migration is not likely to be a significant source of costs for states choose to adopt the expansion.

The limitations of this study should be acknowledged. First, using education as a proxy for income may include individuals being eligible for treatment who are not actually treated by the policy, which leads to downward bias in the point estimate of interest. Second, undocumented immigrants are not eligible for these benefits. However, like other official datasets representing the U.S. population, the ACS does not include data on legal status of immigrants. Although the “residual methodology” we employed allows us to determine with a high likelihood that a given respondent was undocumented, we could not do so with certainty. Thus, we can only estimate an estimate on the overall low-educated non-citizens, which include both documented and undocumented immigrants. Third, state's decision in expanding Medicaid might be correlated with its economic conditions, which may also affect migration and raise endogenous issues. Nevertheless, we find no violation of the parallel trend assumption and the results hold when we include several state-level economic variables.

## Conclusion

We find that Medicaid expansion was not associated with migration to expansion states or out of non-expansion states among the low-educated non-citizens. Having concluded from the evidence that there are no overall systematic migration effects of Medicaid expansion on the overall low-educated non-citizens, we drill down the analysis further, studying movements near state borders, recently unemployed individuals, childless adults, those within 5 to 10 years of immigration, and single men. Overall, estimates in these subsamples are generally consistent with our main findings which show no evidence of welfare-induced migration. In short, the variation across states in accessing Medicaid coverage did not motivate low-educated non-citizens to move to expansion states to pursue public health benefits, and it did not persuade those in expansion states who might have considered leaving to stay put.

## Data availability statement

Publicly available datasets were analyzed in this study. This data can be found here: https://usa.ipums.org/usa/.

## Author contributions

MZ contributed to design of the study and wrote the first draft of the manuscript. HG organized the database and performed the statistical analysis. Both authors contributed to the article and approved the submitted version.

## Funding

This study was supported and funded by the National Social Science Foundation of China (Grant No. 597). Research on the Mechanism, Effect and Policy of the Impact of Labor Migration Rigidity on the Development of Urban-rural Integration in China and the Jiangsu Provincial Social Science Foundation of China (Grant No. 22ZDA001). Research on the Mechanism of Labor Migration Promoting Urban-rural Integration in Jiangsu Province under the Goal of Common Prosperity.

## Conflict of interest

The authors declare that the research was conducted in the absence of any commercial or financial relationships that could be construed as a potential conflict of interest.

## Publisher's note

All claims expressed in this article are solely those of the authors and do not necessarily represent those of their affiliated organizations, or those of the publisher, the editors and the reviewers. Any product that may be evaluated in this article, or claim that may be made by its manufacturer, is not guaranteed or endorsed by the publisher.

## Footnotes

^1^U.S. Department of Health and Human Services, Office of the Assistant Secretary for Planning and Evaluation, 2021 Poverty Guidelines. Available online at: https://aspe.hhs.gov/poverty-guidelines.

^2^Hawaii is an expansion state; Alaska expanded in 2015.

^3^Response rate during our sample period (2010–2017) is consistently over 90 percent. https://www.census.gov/acs/www/methodology/sample-size-and-data-quality/response-rates/.

^4^The Annual Social and Economic Supplement of the Current Population Survey (March CPS) is similar to the ACS in that it asks individuals whether their residence in the previous year was in the same state as their current residence. However, sample size of the March CPS is only about one-third of the ACS. Therefore, we rely on the ACS dataset.

^5^Meyer (14) criticizes the use of sample conditioning on below an income threshold in welfare migration literature, because income usually increases when people migrate from a low benefit state to a high benefit state. This would lead to a bias against finding evidence of welfare migration. Instead, he suggests using the “at-risk group” to study the welfare migration.

^6^Adults over the age of 65 are generally be covered by Medicare.

^7^We probe the robustness of our results by restricting the sample to those with less than five years of residency. Estimates remain largely unchanged. Results are not reported but available upon request.

^8^It is worth noting that immigrants who are naturalized are excluded in our main sample because they are accorded the same access to public benefits as native-born citizens and are more assimilated, meaning their options of benefit use are more similar to those of native-born citizens (15). Buchmueller et al. (16) argue that naturalized citizens “look like” natives. Ku and Matani (17) find that difference in insurance rates between natives and naturalized citizens is not significant, while the percentage of noncitizens who were uninsured was much higher than that of natives. Therefore, we believe separating noncitizen immigrants from naturalized citizens gives a clearer picture of the effects of Medicaid expansion on immigrants.

^9^We call these five states plus DC as “early expanders”.

^10^To assess whether including states with pre-ACA expansions, either limited or comprehensive, made a difference, we re-estimate all models in several ways. First, we drop MA. Second, we drop the “early expanders”. Third, we drop the “early expanders” plus MA. None of specifications yield result that are meaningfully different from the baseline specification.

^11^We present the descriptive statistics with the ACS survey weights in Appendix Table A1.

^12^We exclude individuals who migrated from and to other countries; therefore, we have a unique sample for in-migration and out-migration analyses.

^13^The private purchases include both employer-sponsored and non-group purchases. Estimates for models that divide the privately insured into those without employer-sponsored insurance are largely consistent with the estimates obtained with employer-sponsored insurance. Also, individuals could report having more than one type of coverage in the ACS, and we allow for this in our analysis. Therefore, the types of coverage are not mutually exclusive.

^14^The regressions are weighted by the ACS sample weights. We also performed same regressions without using the sample weights. All estimates are consistent with the main findings. The results are not shown but are available upon request.

^15^We are grateful to Dr. Robert G. Valletta at Federal Reserve Bank of San Francisco for providing us with data on unemployment insurance benefit durations.

^16^FPL is based on each family's total income for previous years as a percentage of the poverty thresholds.

^17^We only present the coefficient for the variable of interest in Table 3. Coefficients of all demographic characteristics are presented in Appendix Table A3.

^18^In response to the 2007-09 “Great Recession,” the maximum duration of unemployment benefits was increased from the normal level of 26 weeks to an unprecedented 99 weeks [see Valletta (28) for details].

^19^This allows us to address concerns regarding potential biased introduced by state-level controls, such as unemployment rate and UI benefits.

^20^We also present results for low-educated natives in (Column 2 of Appendix Table A4) and low-educated naturalized citizens (Column 3 of Appendix Table A4). The estimates are in line with the published evidence that Medicaid expansion was not associated with changes in interstate migration.

^21^One concern of using PUMA as local area is that the PUMA classification system changed in 2012. It is thus possible that the composition of individuals in PUMAs within a certain distance of a state border could have changed substantially between 2010 and 2012. Therefore, we re-estimate our border samples by using 2012-2017 data. Regression estimates are largely unchanged. Results are not reported but available upon request.

^22^One concern of restricting the sample to border PUMAs is it substantially decreases the number of available observations and may result in statistical power issues.

^23^In in-migration equation, we use unemployed at the time of survey; while, in out-migration equation we use unemployed in previous year.

^24^Medicaid eligibility income thresholds for parents who were not eligible for specific Medicaid programs such as the Aged, Blind and Disabled (ABD) program ranged from 16% to 215% of the FPL, with an average of 87.33% of the FPL as of January 2013. However, thresholds for nonparent adults who were not eligible for coverage under the ABD program averaged 18.55% as of January 2013 (1).

^25^We use cross-state migration as our outcome variables under this specification. We cannot use cross expansion/non-expansion migration because the set of expansion states gets larger and the set of non-expansion states gets smaller along with more states counted as expansion states. Mechanically, this decreases in-migration rate in the set of expansion states and increases in-migration rate in the set of non-expansion states.

^26^Additionally, the net premium paid on plans purchased through HIX is positive. Imagine a family of four earning at 138% FPL, which is about $32,000 per year. The insurance premium costs $16,000/ year (which is based on the average cost of 2nd lowest silver plan). Because of the premium subsidies, their insurance premium is capped at 2.04% of their income, and therefore, the maximum the family needs to pay is $653.

^27^Following Borjas (33), we define a person in the United States as legal if the person meets any of the following criteria: (1) arrived in the United States before 1980; (2) was born in Cuba; (3) has U.S. citizenship; (4) receives public benefits; (5) is employed with the government sector; (6) is employed with occupations that require licensing; (7) is married to a legal immigrant or a U.S. citizen. The residual group of all other foreign-born individuals is then classified as “potentially unauthorized” immigrants.

^28^Interpreting in-migration response as an indication of Medicaid-induced migration requires caution in this context, however. This is because such method requires selecting on the basis of Medicaid receipt which could introduce compositional issues.

^29^States extended Medicaid to pregnant women and child immigrants during our study period. In 2010, 26 states provided Medicaid to pregnant immigrants and 24 states to child immigrants. By 2017, the number increased to 32 for both pregnant immigrants and child immigrants (34).

^30^2014 actuarial report: On the financial outlook for Medicaid. Report to Congress. Department of Health and Human Services, 2014.

## References

[1] Henry J Kaiser Family Foundation. Health Coverage of Immigrants. (2021). Available online at: https://www.kff.org/racial-equity-and-health-policy/fact-sheet/health-coverage-of-immigrants/

[2] Borjas GJ. Immigration and welfare magnets. J Labor Econ. (1999) 17:607–37. 10.1086/209933

[3] McKinnish T. Importing the poor: welfare magnetism and cross-border welfare migration. J Hum Resour. (2005) 40:57–76. 10.3368/jhr.XL.1.57 21949446 PMC3177304

[4] McKinnish T. Welfare-induced migration at state borders: new evidence from MicroData. J Public Econ. (2007) 91:437–50. 10.1016/j.jpubeco.2006.09.002 21949449 PMC3177299

[5] Schwartz AL Sommers BD. Moving for medicaid? Recent eligibility expansions did not induce migration from other states. Health Aff. (2014) 33:88–94. 10.1377/hlthaff.2013.0910 24395939

[6] Alm J Enami A. Do government subsidies to low-income individuals affect interstate migration? Evidence from the massachusetts health care reform. Reg Sci Urban Econ. (2017) 66:119–31. 10.1016/j.regsciurbeco.2017.06.005

[7] Yasenov VI Lawrence D Mendoza FS Hainmueller J. Public health insurance expansion for immigrant children and interstate migration of low-income immigrants. JAMA Pediatr. (2020) 174:22–8. 10.1001/jamapediatrics.2019.4241 31738388 PMC6865314

[8] Federation for American Immigration Reform. The Sinking Lifeboat: Uncontrolled Immigration and the U.S. Health Care System in 2009. Washington, DC: Federation for American Immigration Reform (2009). Available online at: https://www.fairus.org/issue/publications-resources/sinking-lifeboat-uncontrolled-immigration-and-us-health-care-system. (accessed July 14, 2022).

[9] Mohanty SA Woolhandler S Himmelstein D Pati S Carrasquillo O Bor D. Health care expenditures of immigrants in the United States: a nationally representative analysis. Am J Public Health. (2005) 95:1431–8. 10.2105/AJPH.2004.044602 16043671 PMC1449377

[10] Goldman DP Smith JP Sood N. Immigrants and the cost of medical care. Health Aff. (2006) 25:1700–11. 10.1377/hlthaff.25.6.1700 17102196

[11] Zallman L Woolhandler S Touw S Himmelstein DU Finnegan KE. Immigrants pay more in private insurance premiums than they receive in benefits. Health Aff. (2018) 37:1663–8. 10.1377/hlthaff.2018.0309 30273017

[12] Sherman A Trisi D Stone C Gonzales S Parrott S. Immigrants Contribute Greatly to U.S. Economy, Despite Administration's “Public Charge” Rule Rationale. Center on Budget and Policy Priorities (2019).

[13] Henry J Kaiser Family Foundation. Status of State Action on the Medicaid Expansion Decision. KFF State Health Facts. (2021). Available online at: https://www.kff.org/health-reform/state-indicator/state-activity-around-expanding-medicaid-under-the-affordable-care-act/

[14] Meyer BD. Do the Poor Move to Receive Higher Welfare Benefits? (2000). Available online at: https://harris.uchicago.edu/files/meyer_do_the_poor.pdf. (accessed on December 25, 2021)

[15] Ku L Bruen B. Poor Immigrants Use Public Benefits at a Lower Rate than Poor Native-Born Citizens. Cato Onstitute Economic Development Bulletin; Center for Global Liberty and Prosperity (2013). Available online at: https://www.cato.org/sites/cato.org/files/pubs/pdf/edb17.pdf

[16] Buchmueller TC Lo Sasso AT Lurie I Dolfin S. Immigrants and employer-sponsored health insurance. Health Serv Res. (2006) 42:286–310. 10.1111/j.1475-6773.2006.00600.x 17355593 PMC1955235

[17] Ku L Matani S. Left out: immigrants' access to health care and insurance. Health Aff. (2001) 20:247–56. 10.1377/hlthaff.20.1.247 11194848

[18] Henry J Kaiser Family Foundation. States Getting a Jump Start on Health Reform's Medicaid Expansion. Washington DC: Kaiser Commission on Medicaid and the Uninsured (2012).

[19] Sommers BD Arntson E Kenney GM Epstein AM. Lessons from early medicaid expansions under health reform: interviews with medicaid officials. Medicaid Res Rev. (2013) 3:4. 10.5600/mmrr.003.04.a02 24834369 PMC4015416

[20] Goodman L. The effect of the affordable care act medicaid expansion on migration. J Policy Anal Manage. (2017) 36:211–38. 10.1002/pam.21952 27992152

[21] Jaeger D. Local Labor Markets, Admission Categories, and Immigrant Location Choice. Hunter College, New York. (2000).

[22] Dodson ME. Welfare Generosity and Location Choices among New United States Immigrants. Int Rev Law Econ. (2001) 2:47–67. 10.1016/S0144-8188(00)00040-5

[23] Kaushal N. New Immigrants' location choices: magnets without welfare. J Labor Econ. (2005) 23:59–80. 10.1086/425433

[24] Kaestner R Garrett B Chen JJ Gangopadhyaya A Fleming C. Effects of ACA medicaid expansions on health insurance coverage and labor supply. J Policy Anal Manage. (2017) 36:608–42. 10.1002/pam.21993 28653821

[25] Courtemanche C Marton J Ukert B Yelowitz A Zapata D. Early impacts of the affordable care act on health insurance coverage in medicaid expansion and non-expansion states. J Policy Anal Manage. (2017) 36:178–210. 10.1002/pam.21961 27992151

[26] Bureau of Labor Statistics. The Local Area Unemployment Statistics. Washington, DC: Bureau of Labor Statistics, (2018). Available online at: http://www.bls/gov/lau/.

[27] Farber HS Valletta RG. Do extended unemployment benefits lengthen unemployment spells? J Hum Resour. (2015) 50:873–909. 10.3368/jhr.50.4.873

[28] Valletta R. G. Recent extensions of U.S. unemployment benefits: search responses in alternative labor market states. IZA J Labor Policy. (2014) 3:1-25 10.1186/2193-9004-3-18

[29] Buchmueller TC Levy H Valletta RG. Medicaid Expansion and the Unemployed. J Labor Econ. (2021) 39:S575–617. 10.1086/712478

[30] Molloy R Smith CL Wozniak A. Internal migration in the United States. J Econ Perspect. (2011) 25:173–96. 10.1257/jep.25.3.173

[31] Barbaresco S Courtemanche C Qi Y. Impacts of the affordable care act dependent coverage provision on health-related outcomes of young adults. J Health Econ. (2015) 40:54–68. 10.1016/j.jhealeco.2014.12.004 25594956

[32] Passel JS Cohn DV. Unauthorized Immigrant Totals Rise in 7 States, fall in 14 States: Decline in Those from Mexico Fuels Most State Decreases. Washington, DC: Pew Research Center. (2014).

[33] Borjas GJ. The labor supply of undocumented immigrants. Labour Econ. (2017) 46:1–13. 10.1016/j.labeco.2017.02.004

[34] Gelatt J Bernstein H Koball H Runes C Pratt E. State Immigration Policy Resource. Urban Institute; February 17. (2022).

