# Supplementary material for: Do non-citizens migrate for welfare benefits? Evidence from the Affordable Care Act Medicaid expansion
Source: Front Public Health. 2022 Sep 30;10:955257. doi: 10.3389/fpubh.2022.955257 (PMC9562776; doi:10.3389/fpubh.2022.955257)
Supplement: Supplementary file 1 [file Data_Sheet_1.pdf]

APPENDIX

Appendix Table A1. Descriptive Statistics (Weighted)

	Non-expansion States		Expansion States	
	Mean	SD	Mean	SD
**Panel A: Demographic Variables**				
Age	40.50	10.91	41.77	10.88
Female	0.45	0.50	0.47	0.50
Married	0.61	0.49	0.59	0.49
Num. of own children	1.59	1.50	1.59	1.46
Hispanic	0.92	0.27	0.89	0.31
Family income as of FPL	162.21	114.62	173.11	119.70
% with income <=138% of the FPL	0.50	0.50	0.46	0.50
**Panel B: Health Insurance Coverage**				
Uninsured	0.71	0.45	0.53	0.50
Employer-sponsored	0.18	0.38	0.21	0.41
Privately Purchased	0.22	0.41	0.24	0.43
Medicaid	0.07	0.25	0.22	0.42
**Panel C: Labor Market**				
In Labor Force	0.72	0.45	0.72	0.45
Unemployed	0.07	0.25	0.09	0.29
Ln (hours)	3.61	0.39	3.59	0.40
Fulltime	0.89	0.32	0.87	0.33
Obs.	109,162		196,224	
**Panel D: In-Migration Rate**				
Cross-state migration	0.0122	0.1098	0.0076	0.0868
Cross E/NE state migration	0.0065	0.0801	0.0025	0.0502
Obs.	109,162		196,224	
**Panel E: Out-Migration Rate**				
Cross-state migration	0.0100	0.0997	0.0089	0.0939
Cross E/NE state migration	0.0043	0.0652	0.0038	0.0618
Obs.	108,898		196,488	

Sources: American Community Survey (2010-2017).

Notes: Sample is limited to noncitizens with less than high school education at ages 18-64. Each cell in panels A-C reports the sample mean of the variable indicated, among noncitizens in the baseline sample with current state in non-expansion states (column 1) or in expansion states (column 2). Panels D and E report the average in-migration and out-migration rate for the studied sample. Cross-state migration means moved across a state border line. Cross E/NE state migration means moved across a state border line between an expansion state and a non-expansion state. FPL is federal poverty level.

**Appendix Table A2.** Changes in Health Insurance Coverage: Income<=138% of the FPL				
	Medicaid	Private-purchased	Employer-sponsored	Uninsured
Expand × post	0.1007***	-0.0446***	-0.0156***	-0.0551***
	(0.0106)	(0.0144)	(0.0046)	(0.0169)
Mean of dep. var. in expansion states before 2014	[0.2504]	[0.1556]	[0.1192]	[0.5972]
State fixed effect and year fixed effect	Yes	Yes	Yes	Yes
Obs.	289,658	289,658	289,658	289,658

Sources: American Community Survey (2010-2017).

Notes: Estimates report coefficients on interaction term between an indicator for whether the state is an expansion state and an indicator for whether the year is after 2014. Sample used in this analysis is limited to noncitizen immigrants between ages 18 and 64 with incomes up to 138% of the FPL. Regressions are adjusted using indictors for state, year, age, age squared, gender, and marital status. State level variables include the unemployment rate and the annual average number of weeks of unemployment insurance benefits available. Regressions are weighted by the ACS sample weights. Mean of dependent variables in expansion states before 2014 are reported in brackets. All standard errors (parentheses) are clustered on current-state level.

* p<0.1; ** p<0.05; *** p<0.01

Appendix Table A3. Changes in in-migration (out-migration) rates for noncitizens

	Model 1		Model 2		Model 3	
	In-migration	Out-migration	In-migration	Out-migration	In-migration	Out-migration
Expand × post14	0.0001	0.0005	-0.0004	0.0003	0.0007	-0.0007
	(0.0010)	(0.0008)	(0.0010)	(0.0008)	(0.0024)	(0.0015)
Age	-0.0004***	-0.0004**	-0.0004***	-0.0004**	-0.0004***	-0.0004**
	(0.0001)	(0.0002)	(0.0001)	(0.0002)	(0.0001)	(0.0002)
Age Squared	0.0000**	0.0000*	0.0000**	0.0000*	0.0000**	0.0000*
	(0.0000)	(0.0000)	(0.0000)	(0.0000)	(0.0000)	(0.0000)
Female	-0.0004	-0.0005*	-0.0004	-0.0005*	-0.0004	-0.0005*
	(0.0004)	(0.0003)	(0.0004)	(0.0003)	(0.0004)	(0.0003)
Married	-0.0014*	-0.0012***	-0.0014*	-0.0012***	-0.0014*	-0.0012***
	(0.0007)	(0.0003)	(0.0007)	(0.0003)	(0.0007)	(0.0003)
UI_weeks			0.0001	0.0001	0.0001	0.0001
			(0.0001)	(0.0001)	(0.0001)	(0.0001)
Unemp. rate			-0.0010**	-0.0002	-0.0003	-0.0003
			(0.0004)	(0.0003)	(0.0010)	(0.0006)
Constant	0.0133***	0.0139***	0.0138*	0.0094*	0.0057	0.0130*
	(0.0025)	(0.0039)	(0.0075)	(0.0053)	(0.0114)	(0.0070)
Demographic controls	Yes	Yes	Yes	Yes	Yes	Yes
State-level controls	No	No	Yes	Yes	Yes	Yes
State linear trends	No	No	No	No	Yes	Yes
Obs.	305,386	305,386	305,386	305,386	305,386	305,386

Notes: Estimates report coefficients of the interaction term of equations (2) and (3). Sample used in this analysis is limited to noncitizen immigrants between ages 18 and 64 with education level less than high school. Regressions are adjusted using indictors for state, year, age, age squared, gender, and marital status. State level variables include the unemployment rate and the unemployment insurance benefit durations. Regressions are weighted by the ACS sample weights. All standard errors (parentheses) are clustered on current-state level for in-migration equations and origin-state level for out-migration equations.

* p<0.1; ** p<0.05; *** p<0.01

Appendix Table A4. Changes in In-migration (Out-migration) Rate for Natives and Naturalized Citizens

	Noncitizen Immigrants		Natives		Naturalized Citizens	
	In-migration	Out-migration	In-migration	Out-migration	In-migration	Out-migration
Expand × post14	-0.0004	0.0003	-0.0004	0.0003	0.0007	-0.0001
	(0.0010)	(0.0008)	(0.0007)	(0.0006)	(0.0015)	(0.0007)
Obs.	305,386	305,386	818,676	818,676	130,695	130,695

Source: American Community Survey (2010-2017).

Notes: Estimates report coefficients of the interaction term of equations (2) and (3). Regressions are adjusted using indictors for state, year, age, age squared, gender, marital status and two state-level variables. Regressions are weighted by the ACS sample weights. All standard errors (parentheses) are clustered on current-state level for in-migration equations and origin-state level for out-migration equations.

* $p<0.1$; ** $p<0.05$; *** $p<0.01$
